# Clinical and histopathological findings of a rare sinonasal glomangiopericytoma

**DOI:** 10.4322/acr.2023.424

**Published:** 2023-04-10

**Authors:** Zahraa Noureddine El Moussaoui, Zahraa Al Najjar, Nada Diab, Zahraa Saker, Hassane Choukr, Ahmad K Aoude, Marwan Saliba, Bilal Shoumar, Mohamad Reda Noureddine El Moussaoui

**Affiliations:** 1 Lebanese University, Faculty of Medical Sciences, Neurology Department, Beirut, Lebanon; 2 Al-Rassoul Al-Aazam Hospital, Pathology Department, Beirut, Lebanon; 3 Al-Rassoul Al-Aazam Hospital, Radiology Department, Beirut, Lebanon; 4 Lebanese University, Faculty of Medical Sciences, Neuroscience Research Center, Beirut, Lebanon; 5 Al-Rassoul Al-Aazam Hospital, Otolaryngology-Head and Neck Surgery Department, Beirut, Lebanon; 6 Al-Rassoul Al-Aazam Hospital, Neurosurgery Department, Beirut, Lebanon; 7 Bahman Hospital, Otolaryngology-Head and Neck Surgery Department, Beirut, Lebanon

**Keywords:** Epistaxis, Nasal Cavity, Paranasal Cavity, Paranasal Sinuses

## Abstract

Glomangiopericytoma is a rare vascular neoplasm of the nasal cavity and paranasal sinuses that occurs during the sixth or seventh decade of life. It is categorized as a borderline tumor with low malignant potential and classified as a distinct entity of sinonasal tumors with perivascular myoid phenotype by the World Health Organization (WHO). We report the case of a 50-year-old woman with nasal obstruction and severe epistaxis. The nasal sinuses computed tomography (CT), and magnetic resonance imaging (MRI) demonstrated a 3.1 cm soft tissue mass occupying the upper part of the left nasal cavity invading the left paranasal sinuses and nasal septum, and the left eye medial rectus muscle. A total mass resection was performed by nasal endoscopy. The histological and immunohistochemical examination yielded the diagnosis of glomangiopericytoma. This case report aims to contribute to the knowledge of nasal neoplasms. The need for more data on this entity is the main obstacle to developing standardized treatment guidelines.

## INTRODUCTION

Glomangiopericytoma, a sinonasal-type hemangiopericytoma, is an uncommon vascular tumor of the nose and paranasal sinuses, accounting for about 0.5% of nose and sinuses neoplasms.^1^ It arises from perivascular-modified glomus-like myoid cells of the sinonasal tract.^2^ The World Health Organization (WHO) has classified this tumor as a distinct entity since it tends to behave more indolently than its soft tissue counterparts.^3^ Even though glomangiopericytoma is considered to have a borderline low malignant and metastatic potential, the recurrence rate was reported to be up to 20% among patients.^2,4^

Glomangiopericytoma can be diagnosed across a broad age range, predominantly in the 6th and 7th decade of life, with a slight female preponderance.^2^ Although its underlying etiology is still unclear, trauma, use of corticosteroids, hypertension, and pregnancy are considered certain risk factors.^5^ The most common symptoms are nasal obstruction, repeated epistaxis episodes, and nonspecific signs such as sinusitis and headache.^6,7^ Endoscopic surgical resection post-embolization or ligation of the feeding arteries is the preferred treatment option due to the vascularized nature of the tumor.^8,9^

We report a case of a 50-year-old female who underwent complete nasal endoscopic excision of glomangiopericytoma. We describe the clinicopathological and surgical characteristics and the radiological examination of this patient.

## CASE REPORT

A 50-year-old female with non-contributory medical history was admitted to the emergency department complaining of nasal obstruction and severe epistaxis of acute onset over the past few days. Under general anesthesia, nasal endoscopic examination revealed the presence of a friable reddish multilobulated mass that filled the left nasal cavity, including a large area of the nasopharynx. A tissue biopsy was performed. The control of the bleeding was achieved by tamponing the nose with surgicel.

**Figure 1:**
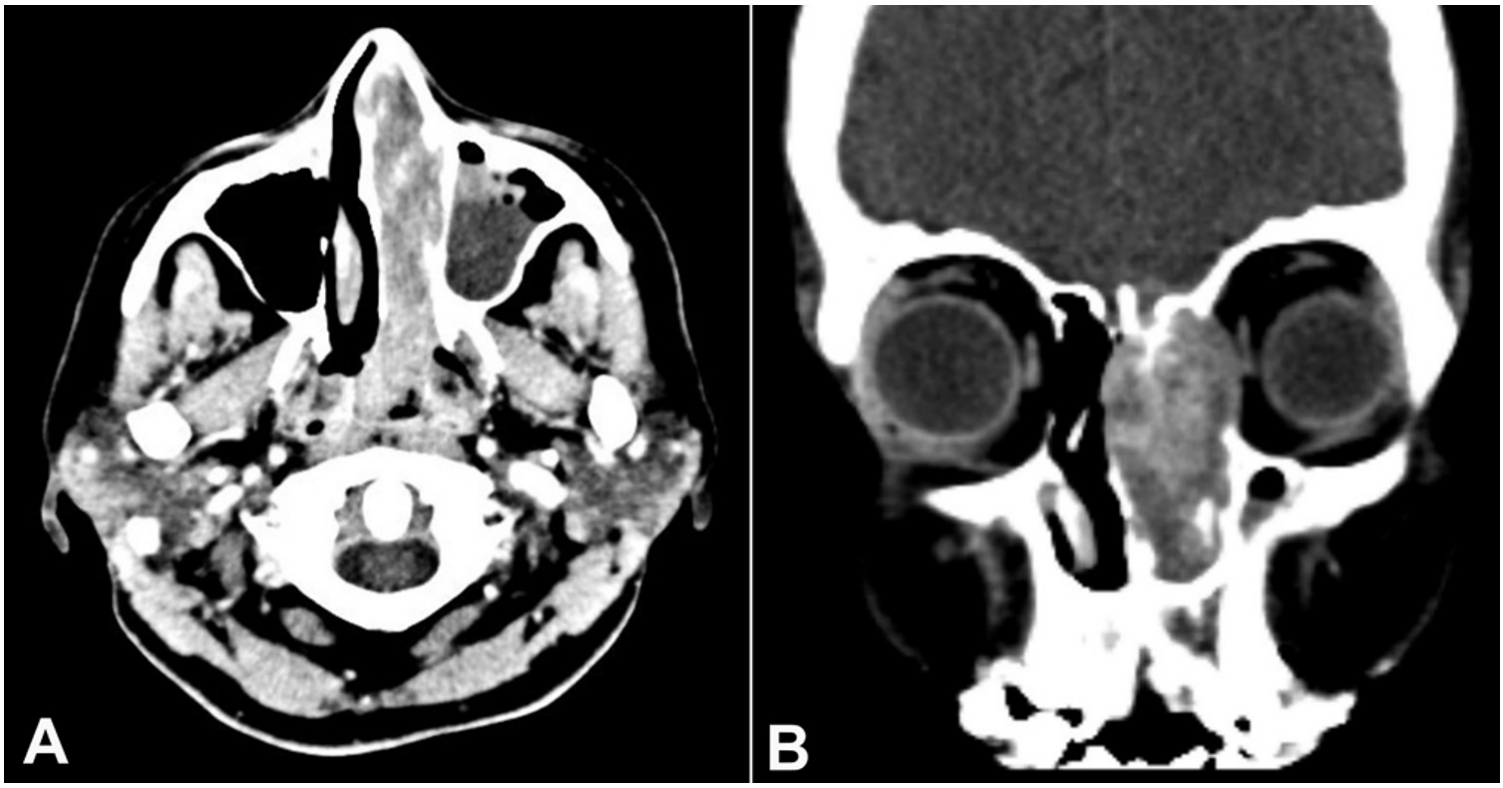
Non-enhanced CT scan showed in: **A -** an expansile soft tissue mass occupying the left nasal cavity, with hyperdensity and extending to the nasopharynx with a significant invasion to the left maxillary sinus; **B -** the mass showed close contact with the medial rectal muscle of the left eye.

An enhanced computed tomography (CT) scan showed the presence of heterogeneous enhanced expansile soft tissue mass occupying the entire left nasal cavity, extending to a large area of the nasopharynx, and invading the left paranasal sinuses, nasal septum, and the medial aspect of the left orbital space with a significant remodeling and multifocal destruction of the surrounding bones. Mild intracranial invasion of the cribriform plate and focal destruction of the cribriform plate of the left ethmoid cells were seen. Remarkably, slight contact between the mass and the left eye medial rectus muscle was noted (Figure 1).

**Figure 2:**
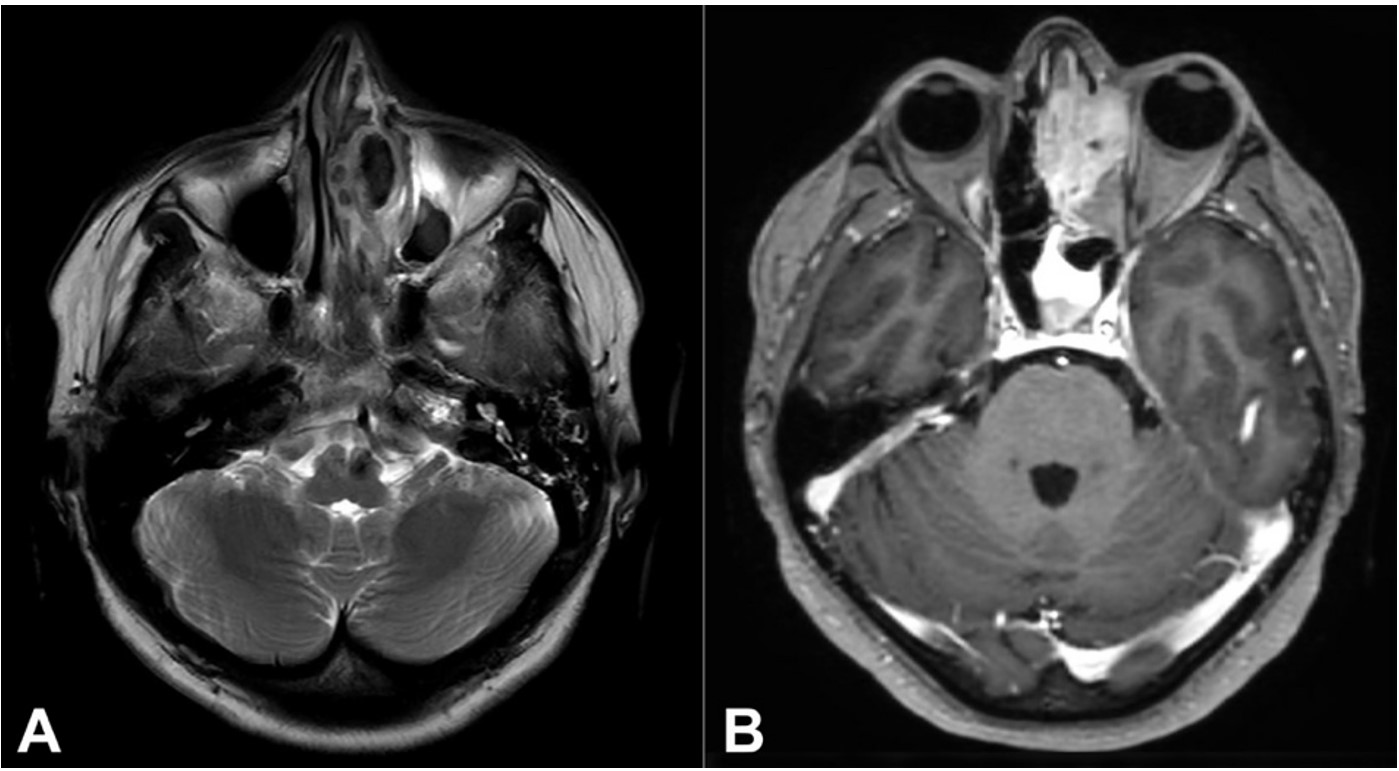
MRI imaging showed: **A -** heterogeneous mass on T2W1; **B -** hemosinus was observed in the left sphenoid sinus.

The magnetic resonance imaging (MRI) revealed a 3.1 x 2.1 x 2.8 cm enhanced mass occupying the upper two-third level of the nasal cavity associated with 1.5 cm hematoma on its inferolateral aspect along with mild hemosinus in the left ethmoid cells, left sphenoid, frontal and maxillary sinuses. The mass showed heterogeneous mild hyperintensity on the T2-weighted image (T2WI), and hypointensity on the T1-weighted image (T1WI), with prominent enhancement after contrast administration (Figure 2). The imaging findings were suggestive of glomangiopericytoma with panhemosinus.

Diffusion-weighted imaging (DWI) TSE at b1000 showed a hyperintense mass with no diffusion restriction. A high signal on the diffusion sequence supported the benignity of this lesion. Notably, all the radiological results were confirmed by two independent radiologists.

**Figure 3:**
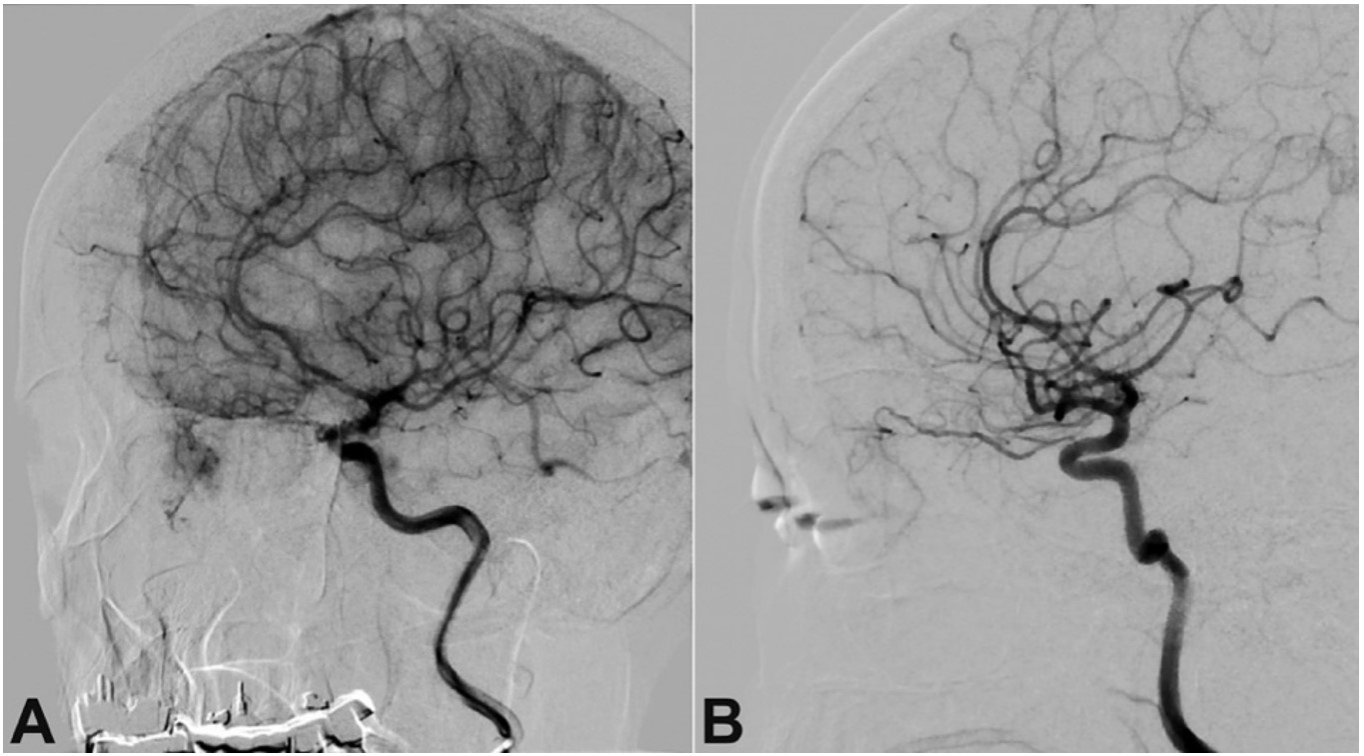
Digital subtraction angiography revealed: **A -** vascular tumor blush in the left nasal cavity; **B -** supplied mainly by the left posterior ethmoidal artery.

Furthermore, cerebral digital subtraction angiography (DSA) for common carotid arteries, internal carotid arteries, and left vertebral arteries was done. Vascular blush in the left nasal cavity compatible with a nasal vascular tumor was seen. The main feeder of the blush was from the left ophthalmic artery branches (anterior and posterior ethmoidal arteries) (Figure 3). Venous drainage through cortical veins, superior sagittal sinus, and both transverse sinuses was normal.

**Figure 4:**
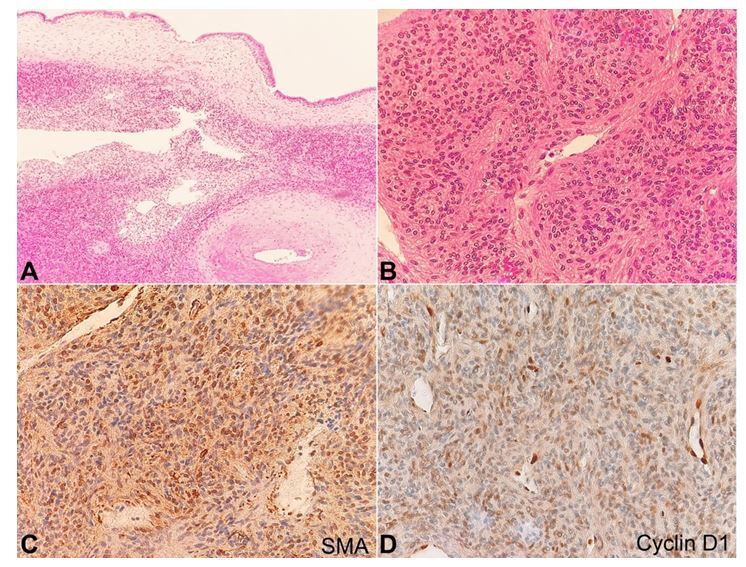
Photomicrographs of the tumor. **A -** subepithelial unencapsulated tumor proliferation separated from the surface of the respiratory epithelium by a grenz zone (H&E, 100X); **B -** glomangiopericytoma cells are bland oval and spindle, arranged in short fascicles around staghorn blood vessels (H&E, 400X); **C -** positive immunohistochemical stainings for smooth muscle actin (400X); **D -** immunohistochemical stainings for cyclin D1 (400X).

**Figure 5:**
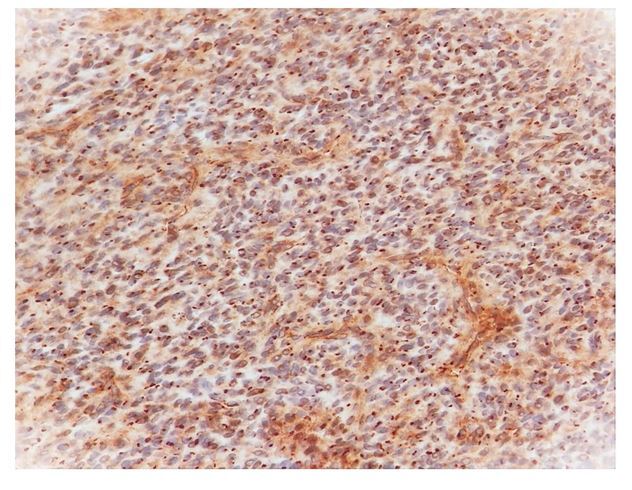
Photomicrograph of the tumor. Positive perinuclear staining for beta-catenin was observed in the majority of tumor cells (x400).

A biopsy was performed and examined by two independent pathologists. Grossly, the mass was beefy reddish to grayish pinky mass with hemorrhage. The histological examination revealed a subepithelial unencapsulated tumor proliferation separated from the surface of the respiratory epithelium by a Grenz zone. The tumor cells appeared as bland oval cells with spindled nuclei and abundant eosinophilic cytoplasm arranged in sheets and small fascicles mainly around ectatic staghorn-type vessels exhibiting perivascular hyalinization (Figure 44B). The stroma was fibro-edematous and myxoid, densified by chronic inflammatory exudates, siderophages, and extravasated red blood cells. Immunohistochemically, the tumor cells were positive to smooth muscle actin, cyclin D1 and beta-catenin (Figures 4C, 4D and Figure 5), but negative to pancytokeratin, synaptophysin, chromogranin, S100, desmine, caldesmon, EMA, STAT6, and CD34. Histological examination, along with immunohistochemical analysis established the diagnosis of sinonasal glomangiopericytoma.

Because of the vascularized nature of the tumor, preoperative ligation of the anterior and posterior ethmoidal arteries was performed. Endoscopic nasal surgery was performed under general anesthesia, where the tumor was entirely removed without bleeding. The canal roof was repaired. The orbital periosteum and the olfactive groove were cleansed to prevent tumor recurrence. The dura was intact and cleared without the complication of cerebrospinal fluid leak.

## DISCUSSION

Glomangiopericytoma was first reported as sinonasal hemangiopericytoma by Stout and Murray.^10^ Then, it was described as hemangiopericytoma-like intranasal tumors by Compagno and Hyams^11^ because of the low malignancy rate in 1976. Later, the WHO defined this tumor as glomangiopericytoma because of its similarity with glomus neoplasms.^3,12^ Remarkably, glomangiopericytoma is usually difficult to be distinguished from other benign vascular tumors such as leiomyoma, angiofibroma, and solitary fibrous tumors.^13^

It is one of the rare nasal tumors, usually present in the nasal cavity and paranasal sinuses, with notably few reported cases in the nasopharynx^2^ as in our case. Our patient’s medical history and clinical presentations did not provide clues for identifying an etiological factor of this tumor. In contrast, some studies have revealed an association between glomangiopericytoma and other diseases such as hypoglycemia,^14^ and oncogenic osteomalacia.^15^

CT, MRI, and DWI showed a soft vascular mass with local invasion. As CT scan and MRI are useful techniques to evaluate tumor invasion and aggression,^16^ cerebral angiography was used to determine the tumor’s vascular supply and to reduce the risk of intraoperative bleeding.^17^ A combination of these findings may be helpful not only to suggest the presence of glomangiopericytoma but also to propose preoperative planning of the surgical approach.^18^

Histological examination by hematoxylin-eosin staining revealed a subepithelial unencapsulated tumor, separated from the respiratory epithelium by the Grenz zone and interspaced with many ectatic staghorn-type vessels. The tumor cells appeared bland oval with spindled nuclei and eosinophilic cytoplasm.^2,16,19^ Sinonasal glomangiopericytoma showed low mitotic activity and atypia, in contrast to the uncommon malignant glomangiopericytoma, which showed high mitotic activity, necrosis nuclear pleomorphism, and bone invasion.^16,20^ Immunohistochemically, glomangiopericytoma can be distinguished from other soft tissue hemangiopericytomas by smooth muscle actin and cyclin D1 positive stainings.^19,21,22^ However, positive reactivity to other markers, such as beta-catenin, CD34, and vimentin, was obtained in many cases.^16,17,21^

Nasal endoscopic excision of glomangiopericytoma with clean margins is considered a less invasive resection method. Chemotherapy and radiotherapy can be used as palliation approaches. However, up to 17% of local recurrence has been discovered as a result of incomplete surgical resection even after along tumor-free intervals.^17,19,23^ Long-term surveillance by means of nasal endoscopy at regular intervals and/or radiological examination should be included as post-operative care.^23^

## CONCLUSION

Glomangiopericytoma is a rare indolent tumor with low malignant potential. The diagnostic approaches include endoscopy, CT, and MRI to define of tumor extent. Angiography is highly recommended to determine the main tumor vascular feeder to reduce bleeding during the operation. Furthermore, histopathological and immunohistochemical analyses are indicated to determine the tumor’s nature, which further helps designate an appropriate treatment approach. Complete surgical resection with regular clinical management and long-term follow-up are required to diagnose possible recurrence.
